# Similar effectiveness of dapagliflozin and GLP‐1 receptor agonists concerning combined endpoints in routine clinical practice: A multicentre retrospective study

**DOI:** 10.1111/dom.13747

**Published:** 2019-05-08

**Authors:** Gian Paolo Fadini, Veronica Sciannameo, Ivano Franzetti, Daniele Bottigliengo, Paola D'Angelo, Carmela Vinci, Paola Berchialla, Salvatore Arena, Raffaella Buzzetti, Angelo Avogaro, for the DARWIN‐T2D network, Agostino Consoli, Gloria Formoso, Giovanni Grossi, Achiropita Pucci, Giorgio Sesti, Francesco Andreozzi, Giuseppe Capobianco, Adriano Gatti, Riccardo Bonadonna, Ivana Zavaroni, Alessandra Dei Cas, Giuseppe Felace, Patrizia Li Volsi, Raffaella Buzzetti, Gaetano Leto, Gian Pio Sorice, Paola D'Angelo, Susanna Morano, Antonio Carlo Bossi, Edoardo Duratorre, Ivano Franzetti, Paola Silvia Morpurgo, Emanuela Orsi, Fabrizio Querci, Massimo Boemi, Federica D'Angelo, Massimiliano Petrelli, Gianluca Aimaretti, Ioannis Karamouzis, Franco Cavalot, Giuseppe Saglietti, Giuliana Cazzetta, Silvestre Cervone, Eleonora Devangelio, Olga Lamacchia, Salvatore Arena, Antonino Di Benedetto, Lucia Frittitta, Carla Giordano, Salvatore Piro, Manfredi Rizzo, Roberta Chianetta, Carlo Mannina, Roberto Anichini, Giuseppe Penno, Anna Solini, Bruno Fattor, Enzo Bonora, Massimo Cigolini, Annunziata Lapolla, Nino Cristiano Chilelli, Maurizio Poli, Natalino Simioni, Vera Frison, Carmela Vinci

**Affiliations:** ^1^ Department of Medicine University of Padova Padova Italy; ^2^ Department of Cardiac, Thoracic and Vascular Sciences, Unit of Biostatistics, Epidemiology and Public Health University of Padova Padova Italy; ^3^ Endocrinology Unit, ASST Valle Olona San Antonio Abate Hospital Gallarate Italy; ^4^ Diabetology Unit Sandro Pertini Hospital, ASLRM 2 Rome Italy; ^5^ Diabetology Unit AULSS 4 Veneto San Donà di Piave Italy; ^6^ Department of Clinical and Biological Sciences University of Turin Turin Italy; ^7^ Unit of Endocrinology and Metabolic Diseases Umberto I Hospital Siracusa Italy; ^8^ Department of Experimental Medicine Sapienza University Rome Italy; ^9^ Università Degli studi G. D'Annunzio di Chieti‐Pescara Dipartimento di Medicina e Scienze dell'Invecchiamento; ^10^ Azienda Sanitaria Provinciale di Cosenza Ospedale San Francesco di Paola; ^11^ Azienda Sanitaria Provinciale di Cosenza; ^12^ Azienda Ospedaliero Universitaria di Catanzaro; ^13^ Azienda Sanitaria Locale Napoli 2 Nord; ^14^ Azienda Sanitaria Locale Napoli 1 Centro Ospedale San Gennaro dei Poveri; ^15^ Azienda Ospedaliero Universitaria di Parma; ^16^ Azienda per l'Assistenza Sanitaria n.5 Friuli Occidentale Ospedale di Spilimbergo; ^17^ Azienda per l'Assistenza Sanitaria n.5 Friuli Occidentale Ospedale di Pordenone; ^18^ Azienda Sanitaria Locale di Latina Ospedale Santa Maria Goretti; ^19^ Fondazione Policlinico Universitario A. Gemelli Roma; ^20^ Azienda Sanitaria Locale Roma 2 Ospedale Sandro Pertini; ^21^ Azienda Ospedaliera Universitaria Policlinico Umberto I Roma; ^22^ Azienda Socio Sanitaria Territoriale Bergamo Ovest Ospedale di Treviglio; ^23^ Ospedale Luini Confalonieri di Luino ‐ Azienda Socio Sanitaria Territoriale Sette Laghi; ^24^ Ospedale Sant'Antonio Abate di Gallarate ‐ Azienda Socio Sanitaria Territoriale Valle Olona; ^25^ Ospedale Fatebenefratelli ‐ Azienda Socio Sanitaria Territoriale Fatebenefratelli Sacco; ^26^ Fondazione IRCCS Ca’ Granda ‐ Ospedale Maggiore Policlinico di Milano; ^27^ Ospedale Pesenti Fenaroli di Alzano Lombardo ‐ Azienda Socio Sanitaria Territoriale Bergamo Est; ^28^ Presidio Ospedaliero di Ricerca INRCA‐IRCCS di Ancona; ^29^ Azienda Ospedaliero Universitaria Ospedali Riuniti di Ancona; ^30^ Azienda Ospedaliero Universitaria Maggiore della Carità di Novara; ^31^ Azienda Ospedaliero Universitaria San Luigi Gonzaga Orbassano; ^32^ Azienda Sanitaria Locale Verbano Cusio Ossola Ospedale Madonna del Popolo di Omegna; ^33^ Azienda Sanitaria Locale di Lecce Casa della Salute, Ugento ‐ Distretto Socio Sanitario Gagliano del Capo; ^34^ Presidio ospedaliero San Marco in Lamis ‐ Distretto Socio Sanitario San Marco in Lamis ‐ Azienda Sanitaria Locale di Foggia; ^35^ Azienda Sanitaria Locale di Taranto Distretto Socio Sanitario di Massafra; ^36^ Azienda Ospedaliero Universitaria Ospedali Riuniti di Foggia; ^37^ Azienda Sanitaria Provinciale di Siracusa Ospedale Umberto I; ^38^ Azienda Ospedaliera Universitaria Policlinico G. Martino di Messina; ^39^ Azienda Ospedaliera di Rilievo Nazionale e di Alta Specializzazione Garibaldi di Catania; ^40^ Azienda Universitaria Policlinico Paolo Giaccone di Palermo; ^41^ Azienda Ospedaliera di Rilievo Nazionale e di Alta Specializzazione Garibaldi di Catania; ^42^ Azienda Universitaria Policlinico Paolo Giaccone di Palermo; ^43^ Azienda USL Toscana Centro Ospedale San Jacopo di Pistoia; ^44^ Azienda Ospedaliero Universitaria Pisana; ^45^ Azienda Ospedaliera Universitaria Pisana; ^46^ Azienda Sanitaria della Provincia Autonoma di Bolzano Comprensorio Sanitario di Bolzano; ^47^ Azienda Ospedaliero Universitaria Integrata di Verona; ^48^ Azienda ULSS n.6 Euganea Complesso Socio Sanitario Ai Colli; ^49^ Azienda ULSS n.9 Scaligera Ospedale Girolamo Fracastoro di San Bonifacio; ^50^ Azienda ULSS n.6 Euganea Ospedale di Cittadella; ^51^ Azienda ULSS n.4 Veneto Orientale

**Keywords:** antidiabetic drug, dapagliflozin, GLP‐1 analogue, glycaemic control, observational study

## Abstract

**Aims:**

According to cardiovascular outcome trials, some sodium‐glucose contransporter‐2 inhibitors (SGLT2i) and glucagon‐like peptide‐1 receptor agonists (GLP‐1RA) are recommended for secondary cardiovascular prevention in type 2 diabetes (T2D). In this real‐world study, we compared the simultaneous reductions in HbA1c, body weight and systolic blood pressure after initiation of dapagliflozin or GLP‐1RA as second or a more advanced line of therapy.

**Materials and methods:**

DARWIN‐T2D was a retrospective multi‐centre study conducted at diabetes specialist clinics in Italy that compared T2D patients who initiated dapagliflozin or GLP‐1RA (exenatide once weekly or liraglutide). Data were collected at baseline and at the first follow‐up visit after 3 to 12 months. The primary endpoint was the proportion of patients achieving a simultaneous reduction in HbA1c, body weight and systolic blood pressure. To reduce confounding, we used multivariable adjustment (MVA) or propensity score matching (PSM).

**Results:**

Totals of 473 patients initiating dapagliflozin and 336 patients initiating GLP‐1RA were included. The two groups differed in age, diabetes duration, HbA1c, weight and concomitant medications. The median follow‐up was 6 months in both groups. Using MVA or PSM, the primary endpoint was observed in 30% to 32% of patients, with no difference between groups. Simultaneous reduction of HbA1c, BP and SBP by specific threshold, as well as achievement of final goals, did not differ between groups. GLP‐1RA reduced HbA1c by 0.3% more than the reduction achieved with dapagliflozin.

**Conclusion:**

In routine specialist care, initiation of dapagliflozin can be as effective as initiation of a GLP‐1RA for attainment of combined risk factor goals.

## 1 INTRODUCTION

The evolution of pharmacotherapy for type 2 diabetes (T2D) has brought about a number of different glucose‐lowering medication (GLM) classes. According to the most recent consensus on the glycaemic management of T2D, the choice of GLM should primarily consider the presence of atherosclerotic cardiovascular disease (CVD) or chronic kidney disease (CKD).^1^ This recommendation is based on the results of cardiovascular outcome trials (CVOTs) that showed improved cardio‐renal outcomes with certain specific sodium‐glucose cotransporter‐2 inhibitors (SGLT2i)^2^, ^3^, ^4^ and glucagon‐like peptide‐1 receptor agonists (GLP‐1RA).^5^, ^6^, ^7^ Although the pattern and type of cardiovascular disease targeted by GLP‐1RAs and SGLT2is appear to be different,^8^ it is remarkable that both classes of GLM have demonstrated cardioprotective effects. Yet, they show different pharmacokinetic properties, as well as differences in efficacy and safety. GLP‐1RAs are injectable and the major adverse events (AEs) are gastrointestinal, while SGLT2i are orally administered and the most frequent AEs are genital infections. Overall, GLP‐1RAs are supposed to be more effective in lowering glucose than are SGLT2is. A head‐to‐head comparative randomized controlled trial (RCT) showed superiority of exenatide once weekly (EOW) vs dapagliflozin when added to metformin monotherapy,^9^ especially in patients with CKD.^10^ However, a network meta‐analysis comparing GLM, added after dual‐therapy failure, suggested no significant difference in the glycaemic effects of GLP‐1RAs and SGLT2is.^11^ In addition to lowering HbA1c, both GLP‐1RAs and SGLT2is significantly improve body weight (BW) and blood pressure (SBP).^12^, ^13^ Such ancillary effects make both drug classes particularly attractive for the comprehensive management of patients with T2D.

Certain combined endpoints have become popular in diabetes phase III RCTs, typically the achievement of HbA1c targets without hypoglycaemia and weight gain.^14^ However, most RCTs continue to focus primarily on glycaemic endpoints or tend to consider BW and BP separately. Yet, since publication of the groundbreaking results of the STENO‐2 study, it has become clear that simultaneously targeting multiple risk factors dramatically improves the micro‐ and macrovascular outcomes.^15^ There is a striking paucity of RCTs that have compared composite outcomes, such as simultaneous reductions in HbA1c, weight and blood pressure, between GLP‐1RAs and SGLT2is. Availability of such comparative assessment could contribute to clinical decision‐making and therapeutic tailoring. Interestingly, change in HbA1c, BW and BP can occur independently; change in HbA1c and reduction in BW are unrelated during therapy with GLP‐1RAs,^15^ whereas reduction in blood pressure is unrelated to glycaemic control during therapy with SGLT2is.^16^

Real‐world evidence from retrospective studies should not substitute for RCTs, but can inform physicians and payers concerning therapeutic effectiveness in clinical practice. Evaluation of surrogate endpoints, such as glycaemia, blood pressure and BW, although correlated with cardiovascular risk, cannot substitute for CVOTs. Nonetheless, several clinically‐relevant questions may remain unanswered, simply because no RCT on that topic is available or planned. For instance, no CVOT is planned to compare SGLT2is and GLP‐1RAs. Thus, in the absence of dedicated RCTs, real‐world data can provide medium‐level evidence to fill such gaps and generate new hypotheses. To address such a gap, we conducted a retrospective real‐world study to compare the effectiveness of the SGLT2i dapagliflozin and GLP‐1RA concerning composite outcomes in clinical practice.

## 2 MATERIALS AND METHODS

### 2.1 Study design

The DARWIN (DApagliflozin Real World evIdeNce)‐T2D trial was a retrospective multi‐centre study conducted at 46 diabetes specialist outpatient clinics in Italy. The protocol was approved by all local ethical committees. The study design and primary results have been published previously.^17^, ^18^ Briefly, the study retrospectively included patients with T2D, as identified in electronic charts, who received first prescriptions for the SGLT2i dapagliflozin, the GLP‐1RA EOW or liraglutide, DPP‐4i (all but linagliptin available) or sulphonylurea (SU) (only gliclazide) between March 2015 and December 2016, according to local practice. In the present analysis, we used only data collected for patients who initiated dapagliflozin, at the full dose of 10 mg, or GLP‐1RA, without having been treated with a member of the same drug class in the past and who continued to use the drug at follow‐up, as recorded in the electronic chart. The study protocol imposed no limitations on background glucose‐lowering therapy, but took into consideration the reimbursement criteria applied in clinical practice. Among SGLT2is, dapagliflozin was chosen because it was the most widely used SGLT2i in Italy when the study was designed and performed. A similar distribution of SGLT2is was observed in other European countries in the CVD‐Real study.^19^ Among GLP‐1RAs, only EOW and liraglutide were included because, when the study began, lixisenatide and exenatide BID were being used in negligible proportions of patients, while dulaglutide and semaglutide were not yet marketed in Italy. The study was initiated before publication of CVOTs concerning SGLT2is and GLP‐1RAs, thereby preventing consideration of cardiovascular protection by specific agents of the two classes.

### 2.2 Data extraction

Dedicated software automatically extracted relevant information from the same electronic chart system (MyStar Connect, Meteda, San Benedetto del Tronto, Italy) at all centres. The following data were collected at baseline (date of first prescription of the above‐mentioned GLMs): age, sex, BW and height to calculate BMI, diabetes duration, systolic and diastolic blood pressure, smoking status, fasting glucose, HbA1c, complete lipid profile, serum creatinine, estimated glomerular filtration rate (eGFR, using the CKD‐EPI equation), urinary albumin excretion rate (in mg/g of creatinine or equivalent), prior and concomitant GLM and other concomitant medications. Based on ICD‐9 codes recorded in the chart, microangiopathy was defined as the presence of one or more of the following: retinopathy (any stage), neuropathy (somatic or autonomic), nephropathy (CKD stage III or higher or micro‐/macroalbuminuria). Macroangiopathy was defined as ischemic heart disease or stroke/transient ischemic attack or peripheral arterial disease or revascularization of coronary, carotid or peripheral arteries.

Updated information was collected concerning HbA1c, BW, BP and medications at the end of follow‐up, which was set as the date of the first visit between 3 and 12 months after baseline. Detailed information concerning drug dosages was not available, but a previous analysis of the DARWIN‐T2D study estimated the final dose of liraglutide to be closer to 1.2 mg than to 1.8 mg.^14^ Dispensing information was also not available.

### 2.3 Definition of endpoints

Three variables were considered in the composite endpoints: change from baseline to end of follow‐up in HbA1c, BW and SBP. The primary endpoint was the proportion of patients achieving a simultaneous reduction in HbA1c, BW and SBP, without thresholds. Thus, to meet the primary endpoint, patients must have had values of HbA1c, BW and SBP at the end of follow‐up that were all lower than the respective values recorded at baseline. Secondary endpoints were (a) the proportion of patients with simultaneous reduction of HbA1c >0.5%, of BW >2 kg and of SBP >2 mm Hg; (b) the proportion of patients simultaneously achieving specific targets at follow‐up: HbA1c ≤7%, BW loss ≥3% and SBP <140 mm Hg; (c) change in the individual components of the composite endpoints.

### 2.4 Power calculation

Based on preliminary data,^17^ we estimated that the proportion of patients meeting the primary endpoint was about 30%. We calculated that, to detect a difference of 10% in the proportion of patients meeting the primary outcome between the two groups, a total of 656 patients would be needed.

### 2.5 Statistical analysis

Continuous variables are described as mean ± standard deviation, unless otherwise specified, whereas categorical variables are presented as percentages. Comparison between two groups was performed with the unpaired two‐tailed Student's *t* test for continuous variables and with the chi‐squared test for categorical variables. Intragroup comparison of the change from baseline to the end of follow‐up in continuous variables was performed using the paired two‐tailed Student's *t* test. Multiple imputation (MI) of missing data was performed using the Multiple Imputation by Chained Equation (MICE) algorithm,^20^ obtaining five imputed datasets. All covariates with less than 40% of missing values were included as predictors in the imputation process, including observed outcome values.^21^ Imputed datasets were used only for multivariable adjustment (MVA) and for calculating propensity scores (PS). Outcome variables were not imputed.

For the MVA approach, logistic regression models were implemented on composite outcomes and linear regression models were built for the change from baseline of HbA1c, BW and SBP. Covariates included clinical characteristics that differed at baseline between the two groups. Non‐normal covariates (eg, triglycerides) according to the Kolmogorov–Smirnov test were log‐transformed. PS were computed in each imputed dataset and the baseline covariates included in the PS models were the following: age, gender, duration of diabetes, BW, BMI, FPG, HbA1c, systolic and diastolic blood pressure, total and HDL cholesterol, triglycerides, eGFR, insulin and metformin therapy, microangiopathy and macroangiopathy.^22^ In a sensitivity analysis, the number of prior GLM drug classes was added as a covariate in the PS models. PS matching (PSM) was performed in each of the five imputed datasets, with the nearest 1:1 ratio without replacement, and with a caliper of 0.15 standard deviations of the distribution of PS on the logit scale.^23^, ^24^ The five matched cohorts varied slightly in composition and size because the five imputed datasets were different and independent. Balance of covariates across the two groups was evaluated using absolute standardized mean differences (STD), using the mean across the five imputed datasets. An STD value below 0.10 was considered suggestive of good balance (ie, difference between groups in continuous variables was <10% of the pooled standard deviation). Outcome analyses were performed in each imputed subset after PSM using the chi‐squared test to compare categorical variables. Estimates of the treatment effect were pooled to obtain the final treatment effect estimate.^25^ To avoid excluding patients from matching, a sensitivity analysis was performed with inverse probability weighting (IPW) to estimate the average treatment effect.^26^ A further adjustment for duration of follow‐up was performed separately for each analysis using logistic regression. Statistical analyses were performed using SPSS ver. 24 or higher and R version 3.4.0 and a two‐tailed *P* value less than 0.05 was considered statistically significant.

## 3 RESULTS

### 3.1 Patient characteristics

**Figure 1:**
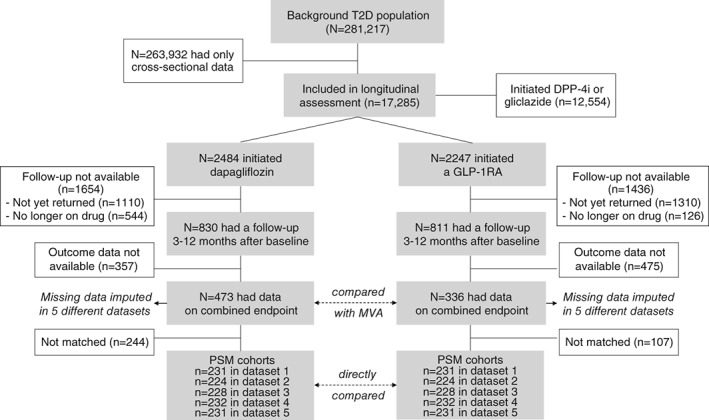
Study flowchart. MVA, multivariable adjustment; PSM, propensity score matching

From a background population of 281 217 patients with T2D, detailed information was retrieved for 17 285 patients who initiated new GLMs. Of these, 2484 initiated dapagliflozin and 2247 initiated a long‐acting GLP‐1RA (EOW or liraglutide). As the main objective of the DARWIN‐T2D trial was to describe baseline patient characteristics, patients did not require a follow‐up visit to be included in the study database. A follow‐up examination was available for 830 patients who initiated dapagliflozin and 811 patients who initiated GLP‐1RA. Reasons for the absence of a follow‐up visit were (i) patients had not yet returned to follow‐up after initiation of new drugs (78%), and (ii) patients discontinued drug use before follow‐up (22%). Information concerning change in HbA1c, BW and SBP for computing the combined endpoints was available at both baseline and follow‐up visits for 473 patients in the dapagliflozin group and for 336 patients in the GLP‐1RA group (Figure 1); the cohort of the present study comprised these patients. The baseline clinical characteristics of these participants are shown in Table 1. As noted previously for the entire study cohort, there were significant differences in many variables between the two groups. Specifically, patients initiating dapagliflozin vs those initiating GLP‐1RA were slightly younger and more often male, with a longer diabetes duration, lower BMI, higher fasting plasma glucose and HbA1c, better renal function, and more frequently were using metformin and insulin. In both groups, patients initiated dapagliflozin or GLP‐1RA after using a median of two (range, one–four) prior GLM classes. There were no significant differences in other concomitant medications and in the complication burden.

**Table 1:** Clinical characteristics of study subjects

	Before PSM				After PSM			
	Dapagliflozin	GLP‐1RA	*P*	D	Dapagliflozin	GLP‐1RA	*P*	D
Number	473	336	‐	‐	231	231	‐	‐
Age, years	59.6 ± 9.4	61.6 ± 9.2	0.003	0.212	60.5 ± 9.1	60.4 ± 9.2	0.899	0.030
Sex male, %	61.1%	54.5%	0.059	0.135	58.4	55.0	0.511	0.014
Diabetes duration, years	11.9 ± 8.1	9.8 ± 7.0	0.001	0.270	10.3 ± 7.7	9.9 ± 6.8	0.542	0.011
BMI, kg/m^2^	33.4 ± 6.0	35.3 ± 5.5	<0.001	0.337	34.7 ± 6.3	34.8 ± 5.6	0.871	0.048
Waist circumference, cm	113.4 ± 13.2	117.6 ± 12.1	0.003	0.336	116.7 ± 14.1	115.5 ± 11.7	0.520	0.039
SBP, mm hg	138.8 ± 18.2	140.6 ± 18.3	0.170	0.098	140.9 ± 18.4	140.0 ± 17.9	0.570	0.009
DBP, mm hg	80.4 ± 10.4	80.5 ± 9.1	0.864	0.012	81.2 ± 9.9	80.3 ± 9.3	0.303	0.004
FPG, mg/dl	171.8 ± 51.3	152.3 ± 32.9	<0.001	0.453	158.9 ± 47.4	153.3 ± 34.3	0.171	0.020
HbA1c, %	8.6 ± 1.4	7.8 ± 0.8	<0.001	0.721	8.0 ± 1.2	7.9 ± 0.9	0.273	0.056
Total cholesterol, mg/dl	171.2 ± 36.4	171.3 ± 41.2	0.976	0.002	174.2 ± 35.8	171.3 ± 42.9	0.487	0.032
HDL cholesterol, mg/dl	45.8 ± 13.4	45.3 ± 11.8	0.622	0.041	46.8 ± 13.4	45.6 ± 12.3	0.371	0.016
Triglycerides, mg/dl	163.8 ± 99.9	164.6 ± 104.6	0.923	0.008	168.8 ± 117.2	162.6 ± 115.1	0.619	0.001
LDL cholesterol, mg/dl	93.3 ± 31.3	92.7 ± 35.3	0.838	0.017	94.5 ± 31.5	93.4 ± 37.4	0.770	0.032
eGFR, mg/min/1.73 m^2^	89.7 ± 15.7	85.8 ± 17.5	0.006	0.232	86.0 ± 16.1	88.7 ± 17.0	0.136	0.009
UAER, mg/24 h	105.0 ± 335.1	103.4 ± 273.0	0.955	0.005	83.5 ± 241.7	103.1 ± 526.3	0.700	0.023
Complications								
Microangiopathy, %	36.3	31.3	0.146	0.105	33.0	28.8	0.385	0.003
Macroangiopathy, %	31.9	32.6	0.853	0.014	34.0	31.6	0.677	0.019
Associated therapy								
Metformin, %	99.4	89.0	<0.001	0.454	98.3	96.5	0.384	0.015
Insulin, %	53.8	21.4	<0.001	0.709	30.9	29.4	0.815	0.015
Prior GLM classes, median (range)^a^	2 (1–4)	2 (1–4)	1.000	0.000	2 (1–4)	2 (1–4)	1.000	0.000
Other therapies								
Anti‐platelet, %	45.7	42.3	0.368	0.068	44.8	42.0	0.634	0.080
Statin, %	64.5	62.0	0.488	0.052	56.1	61.8	0.277	0.017
ACE/ARBs, %	73.3	72.7	0.842	0.015	75.0	74.4	0.882	0.065
CCB, %	23.1	27.7	0.163	0.105	26.4	27.5	0.882	0.023
Beta blockers, %	31.9	32.0	0.978	0.002	33.0	30.0	0.568	0.021
Alpha blockers, %	7.1	9.0	0.363	0.070	7.1	5.9	0.596	0.049
Diuretics, %	10.7	13.0	0.346	0.071	11.3	12.6	0.667	0.012

*Note*: Data are presented for the entire cohort before propensity score matching (PSM) and after PSM. For matched groups, representative data are shown for the first imputed dataset, whereas *P* values and standardized difference (D) are shown for all imputed datasets pooled together. Only observed data are shown.

a To compute the number of GLM classes, the following classes were considered: insulin, metformin, classic secretagogues (sulphonylureas and repaglinide), dipeptidyl peptidase‐4 inhibitors, glitazones (only pioglitazone was available), acarbose.

Abbreviations: ACEi, angiotensin converting enzyme inhibitors; ARBs, angiotensin receptor blockers; BDP, diastolic blood pressure; BMI, body mass index; CCB, calcium channel blockers; eGFR, estimated glomerular filtration rate; FPG, fasting plasma glucose; GLM, glucose‐lowering medications; HDL, high‐density cholesterol; LDL, low‐density cholesterol; SBP, systolic blood pressure; UAER, urinary albumin excretion rate.

As the combined endpoint was available for approximately half of the patients with a follow‐up examination, we evaluated selection bias by comparing patients with and without endpoint data (Table S1). These two groups differed significantly in terms of fasting glucose, total and LDL cholesterol, eGFR, and concomitant use of insulin and ACE inhibitors or angiotensin receptor blockers.

### 3.2 Changes in concomitant medications

As expected in routine clinical practice, some patients underwent changes in concomitant medication at the time they initiated dapagliflozin or a GLP‐1RA (Figure S1). Patients initiating dapagliflozin had a slightly more frequent initiation of insulin and a prescription for sulphonylureas/repaglinide and pioglitazone than patients initiating GLP‐1RAs. On the other hand, patients initiating GLP‐1RAs were more frequently switching from a DPP‐4i‐based regimen. Minor changes were noted for BP‐ and BW‐lowering drugs.

### 3.3 Analyses of effectiveness

**Figure 2:**
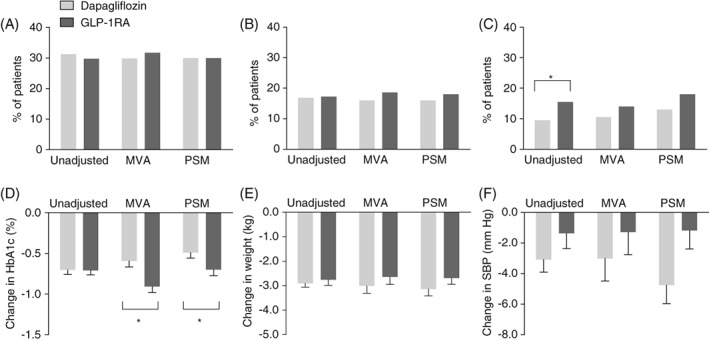
Comparative effectiveness concerning combined and individual endpoints. The proportion of patients in the unadjusted, multivariable adjusted (MVA) and propensity score‐matched (PSM) analyses attaining the primary combined endpoint of any reduction in HbA1c, body weight and systolic blood pressure (A); the combined endpoint of reduction in HbA1c >0.5%, body weight >2 kg and systolic blood pressure >2 mm Hg (B); or the composite target of final HbA1c ≤7.0%, body weight loss ≥3% and systolic blood pressure <140 mm Hg (C); change from baseline to the end of follow‐up in HbA1c (D), body weight (E) and systolic blood pressure (F) in the unadjusted, MVA and PSM analyses. *P < 0.05 for the indicated comparison. The histograms in panels D through F indicate mean and SEM

Median (IQR) duration of follow‐up was 5.9 (4.0–6.5) months in the dapagliflozin group and 6.0 (4.4–6.6) months in the GLP‐1RA group. Table 2 shows outcome analyses according to different statistical approaches. The observed percentage of patients achieving the primary endpoint of any reduction in HbA1c, BW and SBP did not differ between the dapagliflozin and the GLP‐1RA groups (31.3% vs 29.8%; *P* = 0.642). After adjusting for confounders, the proportion of patients attaining the primary endpoint also did not differ between groups, and the odds ratio (OR) was 0.91 (95% CI, 0.64–1.30; *P* = 0.631) (Figure 2A) for dapagliflozin vs GLP‐1RA. Similarly, the percentage of patients achieving a reduction in HbA1c greater than 0.5%, in BW greater than 2 kg and in SBP greater than 2 mm Hg in unadjusted and MVA analyses did not differ between groups (OR, 0.82; 95% CI, 0.53–1.27; *P* = 0.397) (Figure 2B). Finally, although more patients in the GLP‐1RA group obtained simultaneously a final HbA1c of 7.0% or less, a BW loss of at least 3% the basal value, and a final SBP lower than 140 mm Hg in unadjusted analysis (15.5% vs 9.5%; *P* = 0.010), this difference disappeared after MVA (Figure 2C).

**Table 2:** Percentages of patients achieving combined endpoints in the two groups

Combined endpoint	Dapagliflozin (n = 473)	GLP‐1RA (n = 336)	*P*	OR
Any reduction in HbA1c, BW and SBP, %				
Unadjusted	31.3	29.8	0.642	1.05 (0.85–1.30)
MVA	29.9	31.7	0.631	0.91 (0.64–1.30)
PSM (average n = 229/group)	30.3	30.2	0.760	0.93 (0.61–1.44)
ΔHbA1c > 0.5%; ΔBW > 2 kg; ΔSBP>2 mm Hg, %				
Unadjusted	16.9	17.3	0.897	0.98 (0.72–1.33)
MVA	16.0	18.6	0.397	0.82 (0.53–1.27)
PSM (average n = 229/group)	16.5	18.2	0.561	0.86 (0.53–1.41)
HbA1c ≤ 7.0%; ΔBW≥3%; SBP <140 mm Hg, %				
Unadjusted	9.5	15.5	0.010	0.61 (0.42–0.89)
MVA	10.5	14.0	0.187	0.71 (0.44–1.15)
PSM (average n = 229/group)	12.6	17.7	0.183	0.70 (0.41–1.19)

*Note*: The three composite endpoints are given and data are reported for unadjusted analysis (percentages observed in the entire cohort), for multivariable adjustment (percentages estimated from regression models) and for propensity score‐matched analysis (percentages observed in matched groups).

*Note*: Multivariable adjustment included the following variables: age, sex, diabetes duration, HbA1c, eGFR, concomitant use of metformin and insulin.

Abbreviations: BW, body weight; BMI, fasting plasma glucose; OR, odds ratio; SBP, systolic blood pressure.

In patients who initiated dapagliflozin, HbA1c declined by 0.7%, BW by 2.9 kg and SBP by 3.1 mm Hg. In patients who initiated GLP‐1RA, HbA1c declined by 0.7%, BW by 2.8 kg and SBP by 1.4 mm Hg. After MVA, HbA1c declined more significantly in the GLP‐1RA group (by 0.32 ± 0.07%; *P* < 0.001) (Figure 2D), whereas changes in BW and SBP did not differ between groups (Figure 2E,F).

As a second approach, we ran PSM and outcome analyses on five imputed datasets. On average, PSM yielded two very well balanced groups (Figure S2), each composed of an average of 229 patients (n = 231; 224; 228; 232; 231 in the five datasets), allowing direct comparison of outcomes (Figure 2). However, several cases could not be matched because of the partial overlap in PS between the two groups (Figure S3). Representative clinical characteristics of the two groups from the first imputed dataset (n = 231) are shown in Table 2. The proportion of patients with any simultaneous decline in HbA1c, BW and SBP did not differ between groups (dapagliflozin vs GLP‐1RA: OR, 0.93; 95% CI, 0.61–1.44; *P* = 0.760) (Figure 2A). The OR for the composite endpoint of reduction in HbA1c greater than 0.5%, in BW greater than 2 kg and in SBP greater than 2 mm Hg was 0.86 (*P* = 0.561) (Figure 2B). Finally, the proportion of patients simultaneously reaching a final HbA1c of 7.0% or less, a BW loss of at least 3% and a final SBP less than 140 mm Hg tended to be lower with dapagliflozin vs GLP‐1RA, but not significantly (*P* = 0.183) (Figure 2C). HbA1c declined more in the GLP‐1RA group, by 0.29% (95% CI, −0.46; −0.12; *P* < 0.001) (Figure 2D), whereas changes in BW and SBP did not differ between groups (Figure 2E,F).

### 3.4 Sensitivity analysis

Although the median number and range of prior GLM classes was superimposable in the two groups, we first performed a sensitivity analysis with prior GLM class number in the PS models because line of therapy could determine the probability of treatment. The resulting cohorts comprised an average of 224 patients per group in the five imputed datasets, which were well matched (mean STD <0.10 for all characteristics). There was no change in the overall study results, with no significant difference between groups in any of the three combined endpoints (Table S2). In the second sensitivity analysis, IPW was performed with or without incorporation of the prior number of GLM classes in the PS and there was no difference in any of the outcomes between the two groups (Table S2). Finally, when analyses were further adjusted for follow‐up duration, which, overall, did not differ between the two groups, results did not change substantially (Table S3).

## 4 DISCUSSION

This observational study shows that, during treatment of T2D within routine specialist care, a similar proportion of patients initiating the SGLT2i dapagliflozin or a GLP‐1RA after a median of two GLM classes reached composite endpoints of simultaneous reductions in HbA1c, BW and SBP. This finding has implications for the care of T2D patients because certain specific SGLT2is and GLP‐1RAs are now recommended for secondary cardiovascular prevention.^1^ As cardiovascular risk in T2D is driven, not only by hyperglycaemia, but also by concomitant risk factors,^19^, ^27^ the ancillary effects of GLM are particularly attractive.

Both SGLT2is and GLP‐1RAs exert favourable extra‐glycaemic effects on risk factors, but these have not yet been formally analysed in RCTs as combined endpoints. In our study, the proportion of patients experiencing a simultaneous reduction in HbA1c, BW and SBP was identical in the dapagliflozin and GLP‐1RA groups. GLP‐1RA was significantly more effective than dapagliflozin in reducing HbA1c by 0.3% in both the MVA (95% CI, 0.2%–0.5%) and PSM (95% CI, 0.1%–0.5%) analyses (Figure 2D), which is, overall, in line with results of the DURATION‐8 trial.^9^ Nonetheless, when specific thresholds were incorporated into the composite endpoint, only marginal non‐significant differences emerged between the two treatments, probably because the effects of dapagliflozin on BW and SBP counterbalanced the larger effects of GLP‐1RA on HbA1c. When the proportion of patients simultaneously reaching specific targets was considered, a trend in favour of GLP‐1RA emerged because of the greater glycaemic effect, although the choice of specific thresholds for endpoint variables may be considered arbitrary. Selection of the primary endpoint may also be disputable, but prioritization of endpoints in observational research is much less important than in RCTs, because real‐world studies are intrinsically hypothesis‐generating and endpoints were not tested hierarchically. As CVOTs have demonstrated the way that achievement of combined targets is associated with better cardiovascular outcomes in T2D,^16^ we speculate that, among patients receiving SGLT2is or GLP‐1RAs, those attaining combined treatment goals may benefit more from the cardiovascular protection conveyed by such drugs. Unlike the populations of CVOTs, however, the population addressed in the present study comprised mainly patients without a history of CVD.

In the DARWIN‐T2D study, there was an extensive channelling of dapagliflozin to difficult‐to‐treat patients.^17^ Even if there was common support between patients who initiated dapagliflozin and those who initiated a GLP‐1RA (Figure S2), there remained important differences between the two groups, including baseline HbA1c and BW. To address this confounding, we alternatively used MVA or PSM, which are two very different statistical approaches. MVA allows using data from all patients but assumes linear relationships between covariates and outcomes. PSM simulates a quasi‐experimental setting, requires no further adjustment, and makes no assumption concerning the relationships between variables that determine PS and the outcome, but restricts the analysis to matched patients. Importantly, the two approaches led to superimposable results for both combined and individual endpoints. We recognize that neither MVA nor PSM can eliminate all biases and, thus, we cannot rule out residual confounding by unmeasured variables, such as diet and exercise habits, as well as patient preference, compliance and socio‐economic status. PSM led to the exclusion of more than 40% of patients because no good match was found. Thus, to include data from all patients, we performed a sensitivity analysis with IPW, which confirmed the findings obtained with MVA and PSM.

Other limitations to the study should be noted. Although patients were extensively characterized and we tried to adjust the analysis for as many clinical variables as possible, because of confounding by unmeasured variables, the results of observational studies are never able to provide the same level of evidence generated by RCTs. We also acknowledge that data missing from the study database were necessarily handled by multiple imputation, thereby increasing the uncertainty of the estimates. Missing data for some outcome variables, which we decided not to impute, led to exclusion of several patients from the analysis, further limiting generalizability. In RCTs, a large part of the treatment effects of SGLT2is and GLP‐1RAs was already evident at 3 to 6 months,^20^, ^21^, ^22^, ^28^ but the relatively short follow‐up period of our study did not provide information concerning whether the similar benefits of the two treatments would persist during a longer follow‐up period. Finally, we wish to emphasize that, when the study was designed, dapagliflozin was the most used SGLT2i, while liraglutide and EOW were the most used GLP‐1RA, and no CVOTs concerning these medication classes were available. Thus, the results apply only to the two GLP‐1RAs included in the analysis, namely EOW and liraglutide, at a dose close to 1.2 mg,^26^ and may not be generalizable to other SGLT2is or other GLP‐1RAs, such as dulaglutide or semaglutide. These findings should be interpreted in view of the different results of CVOTs, some of which have been incorporated into medication labels. Unlike liraglutide and semaglutide,^6^, ^7^ EOW did not meet the primary superiority endpoint.^5^ Some differences in cardiovascular protective effects emerged also among SGLT2is, probably explained by the different populations investigated.^27^

Notwithstanding these limitations, our data provide valuable information for the comparative assessment of the effectiveness of SGLT2is and GLP‐1RAs on combined endpoints that are important for cardiovascular protection. The concept of “STENO‐2 in a pill” favours a more widespread use of combined endpoints of multiple risk factor targets when evaluating GLMs in RCTs and in the real world. Our study shows that initiation of dapagliflozin can be as effective as initiation of a GLP‐1RA in simultaneously reducing HbA1c, BW and SBP within routine specialist care. Dedicated RCTs are needed to challenge or confirm this finding.

## CONFLICT OF INTEREST

G. P. F. received grant support and lecture or advisory board fees from AstraZeneca, Boehringer‐Ingelheim, Eli Lilly, NovoNordisk, Sanofi, Genzyme, Abbott, Novartis and Merck Sharp & Dohme. R. B. received lecture or advisory board fees from Sanofi, Abbott, Lilly and Astrazeneca. A. A. received research grants and lecture or advisory board fees from Merck Sharp & Dome, AstraZeneca, Novartis, Boeringher‐Ingelheim, Sanofi, Mediolanum, Janssen, NovoNordisk, Lilly, Servier and Takeda. V. S., D. B., I. F., C. V., P. B., P. D. and S. A. declare no conflicts of interest.

## AUTHOR CONTRIBUTIONS

G. P. F., V. S., I. F., D. B., P. B., P. D., C. V., S. A., R. B. and A. A. were responsible for study design, data collection and analysis. G. P. F., V. S. and A. A. were responsible for writing the manuscript. G. P. F., V. S., I. F., D. B., P. B., P. D., C. V., S. A., R. B. and A. A. were responsible for revising the manuscript for intellectual content. All authors approved the final version of the manuscript.

### Composition of the DARWIN‐T2D network

Agostino Consoli and Gloria Formoso (Dipartimento di Medicina e Scienze dell'Invecchiamento ‐ Università Degli studi G. D'Annunzio di Chieti‐Pescara); Giovanni Grossi (Ospedale San Francesco di Paola ‐ Azienda Sanitaria Provinciale di Cosenza); Achiropita Pucci (Azienda Sanitaria Provinciale di Cosenza); Giorgio Sesti and Francesco Andreozzi (Azienda Ospedaliero Universitaria di Catanzaro); Giuseppe Capobianco (Azienda Sanitaria Locale Napoli 2 Nord); Adriano Gatti (Ospedale San Gennaro dei Poveri ‐ Azienda Sanitaria Locale Napoli 1 Centro); Riccardo Bonadonna, Ivana Zavaroni and Alessandra Dei Cas (Azienda Ospedaliero Universitaria di Parma); Giuseppe Felace (Ospedale di Spilimbergo ‐ Azienda per l'Assistenza Sanitaria n.5 Friuli Occidentale); Patrizia Li Volsi (Ospedale di Pordenone ‐ Azienda per l'Assistenza Sanitaria n.5 Friuli Occidentale); Raffaella Buzzetti and Gaetano Leto (Ospedale Santa Maria Goretti ‐ Azienda Sanitaria Locale di Latina); Gian Pio Sorice (Fondazione Policlinico Universitario A. Gemelli, Roma); Paola D'Angelo (Ospedale Sandro Pertini ‐ Azienda Sanitaria Locale Roma 2); Susanna Morano (Azienda Ospedaliera Universitaria Policlinico Umberto I, Roma); Antonio Carlo Bossi (Ospedale di Treviglio ‐ Azienda Socio Sanitaria Territoriale Bergamo Ovest); Edoardo Duratorre (Ospedale Luini Confalonieri di Luino ‐ Azienda Socio Sanitaria Territoriale Sette Laghi); Ivano Franzetti (Ospedale Sant'Antonio Abate di Gallarate ‐ Azienda Socio Sanitaria Territoriale Valle Olona); Paola Silvia Morpurgo (Ospedale Fatebenefratelli ‐ Azienda Socio Sanitaria Territoriale Fatebenefratelli Sacco); Emanuela Orsi (Fondazione IRCCS Ca’ Granda ‐ Ospedale Maggiore Policlinico di Milano); Fabrizio Querci (Ospedale Pesenti Fenaroli di Alzano Lombardo ‐ Azienda Socio Sanitaria Territoriale Bergamo Est); Massimo Boemi† and Federica D'Angelo (Presidio Ospedaliero di Ricerca INRCA‐IRCCS di Ancona); Massimiliano Petrelli (Azienda Ospedaliero Universitaria Ospedali Riuniti di Ancona); Gianluca Aimaretti and Ioannis Karamouzis (Azienda Ospedaliero Universitaria Maggiore della Carità di Novara); Franco Cavalot (Azienda Ospedaliero Universitaria San Luigi Gonzaga, Orbassano); Giuseppe Saglietti† (Ospedale Madonna del Popolo di Omegna ‐ Azienda Sanitaria Locale Verbano Cusio Ossola); Giuliana Cazzetta (Casa della Salute, Ugento ‐ Distretto Socio Sanitario Gagliano del Capo ‐ Azienda Sanitaria Locale di Lecce); Silvestre Cervone (Presidio ospedaliero San Marco in Lamis ‐ Distretto Socio Sanitario San Marco in Lamis ‐ Azienda Sanitaria Locale di Foggia); Eleonora Devangelio (Distretto Socio Sanitario di Massafra ‐ Azienda Sanitaria Locale di Taranto); Olga Lamacchia (Azienda Ospedaliero Universitaria Ospedali Riuniti di Foggia); Salvatore Arena (Ospedale Umberto I – Azienda Sanitaria Provinciale di Siracusa); Antonino Di Benedetto (Azienda Ospedaliera Universitaria Policlinico G. Martino di Messina); Lucia Frittitta (Azienda Ospedaliera di Rilievo Nazionale e di Alta Specializzazione Garibaldi di Catania); Carla Giordano (Azienda Universitaria Policlinico Paolo Giaccone di Palermo); Salvatore Piro (Azienda Ospedaliera di Rilievo Nazionale e di Alta Specializzazione Garibaldi di Catania); Manfredi Rizzo, Roberta Chianetta and Carlo Mannina (Azienda Universitaria Policlinico Paolo Giaccone di Palermo); Roberto Anichini (Ospedale San Jacopo di Pistoia – Azienda USL Toscana Centro); Giuseppe Penno (Azienda Ospedaliero Universitaria Pisana); Anna Solini (Azienda Ospedaliera Universitaria Pisana); Bruno Fattor (Comprensorio Sanitario di Bolzano ‐ Azienda Sanitaria della Provincia Autonoma di Bolzano); Enzo Bonora and Massimo Cigolini (Azienda Ospedaliero Universitaria Integrata di Verona); Annunziata Lapolla and Nino Cristiano Chilelli (Complesso Socio Sanitario Ai Colli ‐ Azienda ULSS n.6 Euganea); Maurizio Poli (Ospedale Girolamo Fracastoro di San Bonifacio ‐ Azienda ULSS n.9 Scaligera); Natalino Simioni and Vera Frison (Ospedale di Cittadella ‐ Azienda ULSS n.6 Euganea); Carmela Vinci (Azienda ULSS n.4 Veneto Orientale).

## Supporting information


**Table S1.** Comparison between patients included in the composite outcome analysis and patients excluded from the analysis for missing outcome information.
**Table S2.** Sensitivity analyses.
**Table S3.** Adjustment for follow‐up duration.
**Figure S1.** Concomitant change in medication prescription.
**Figure S2.** Rebalancing of patient characteristics after propensity score matching.
**Figure S3.** Common support between the two groups of patients.
