# Supplementary material for: Similar effectiveness of dapagliflozin and GLP‐1 receptor agonists concerning combined endpoints in routine clinical practice: A multicentre retrospective study
Source: Diabetes Obes Metab. 2019 May 8;21(8):1886–94. doi: 10.1111/dom.13747 (PMC6767088; doi:10.1111/dom.13747)
Supplement: Supplementary file 1 — Table S1. Comparison between patients included in the composite outcome analysis and patients excluded from the analysis for missing outcome information. Table S2. Sensitivity analyses. Table S3. Adjustment for follow‐up duration. Figure S1. Concomitant change in medication prescription. Figure S2. Rebalancing of patient characteristics after propensity score matching. Figure S3. Common support between the two groups of patients. [file DOM-21-1886-s001.docx]

**Table S1**. Comparison between patients included in the composite outcome analysis and patients excluded from the analysis for missing outcome information. BMI, body mass index. SBP, systolic blood pressure. BDP, diastolic blood pressure. FPG, fasting plasma glucose. HDL, high-density cholesterol. LDL, low-density cholesterol. eGFR, estimated glomerular filtration rate. UAER, urinary albumin excretion rate. ACEi, angiotensin converting enzyme inhibitors. ARBs, angiotensin receptor blockers. CCB, calcium channel blockers.

|  | **Excluded** | | **Included** | | **Comparison** | |
| --- | --- | --- | --- | --- | --- | --- |
|  | **% available** | **Value** | **% available** | **Value** | **p** | **D** |
| **Number** |  | 809 |  | 832 |  |  |
| **Age, years** | 100.0 | 61.4±9.1 | 100.0 | 60.4±9.3 | 0.039 | 0.10 |
| **Sex male, %** | 100.0 | 57.0 | 100.0 | 58.3 | 0.584 | 0.03 |
| **Diabetes duration, years** | 100.0 | 11.4±7.8 | 100.0 | 11.0±7.7 | 0.259 | 0.06 |
| **BMI, kg/m^2^** | 83.3 | 34.1±6.0 | 97.7 | 34.2±5.9 | 0.711 | 0.02 |
| **Waist circumference, cm** | 30.1 | 112.7±12.7 | 38.3 | 115.4±12.9 | 0.013 | 0.21 |
| **SBP, mm Hg** | 23.7 | 138.8±19.4 | 100.0 | 139.5±18.3 | 0.608 | 0.04 |
| **DBP, mm Hg** | 23.7 | 81.8±10.7 | 99.9 | 80.4±9.9 | 0.090 | 0.13 |
| **Fasting glucose, mg/dl** | 60.9 | 171.0±49.6 | 92.1 | 163.9±45.7 | 0.009 | 0.15 |
| **HbA1c, %** | 85.6 | 8.2±1.2 | 100.0 | 8.2±1.2 | 0.161 | 0.07 |
| **Total cholesterol, mg/dl** | 50.0 | 178.7±41.5 | 75.9 | 171.3±38.5 | 0.004 | 0.18 |
| **HDL cholesterol, mg/dl** | 48.1 | 45.1±12.7 | 74.5 | 45.6±12.7 | 0.514 | 0.04 |
| **Triglycerides, mg/dl** | 49.6 | 174.2±127.0 | 75.8 | 164.1±101.8 | 0.159 | 0.09 |
| **LDL cholesterol, mg/dl** | 46.3 | 99.3±33.9 | 72.9 | 93.0±33.0 | 0.004 | 0.19 |
| **eGFR, ml/min/1.73 m2** | 25.2 | 82.2±17.8 | 56.7 | 87.0±16.6 | <0.001 | 0.27 |
| **UAER, mg/g** | 21.6 | 99.5±281.2 | 35.7 | 108.1±328.4 | 0.772 | 0.03 |
| **Associated therapy** | 99.9 |  | 99.9 |  |  |  |
| **Insulin, %** |  | 91.2 |  | 95.2 | 0.001 | 0.16 |
| **Metformin, %** |  | 39.4 |  | 40.5 | 0.665 | 0.02 |
| **Other therapies** | 71.7 |  | 89.0 |  |  |  |
| **Anti-platelet, %** |  | 44.3 |  | 44.3 | 0.991 | 0.00 |
| **Statin, %** |  | 61.4 |  | 63.5 | 0.451 | 0.04 |
| **ACEi/ARBs, %** |  | 66.5 |  | 73.1 | 0.010 | 0.14 |
| **CCB, %** |  | 22.7 |  | 25.0 | 0.337 | 0.05 |
| **Beta-blockers, %** |  | 30.0 |  | 31.9 | 0.441 | 0.04 |
| **Diuretics, %** |  | 10.3 |  | 11.7 | 0.421 | 0.04 |
| **Complications** |  |  |  |  |  |  |
| **Microangiopathy, %** | 88.4 | 36.7 | 98.3 | 34.2 | 0.301 | 0.05 |
| **Macroangiopathy, %** | 80.3 | 29.5 | 88.4 | 32.2 | 0.279 | 0.06 |

**Table S2.** Sensitivity analyses. ^1^In the first sensitivity analysis, the number of prior glucose lowering medication (GLM) classes was included in the propensity score (PS) model to perform PS matching (PSM). ^2^In the second sensitivity analysis, we used inverse probability weighting (IPW) to estimate the average treatment effect with or without incorporation of the prior number of GLM classes in the PS.

In both analyses, the pooled OR (with 95% C.I.) for each composite endpoint was obtained from the 5 imputed datasets and calculated for patients who received dapagliflozin versus those who received GLP-1RA.

| **Outcome** | **PSM^1^** | **IPW^2^** |
| --- | --- | --- |
| **Any reduction in HbA1c, BW, and SBP** |  |  |
| **Without prior GLM classes** | 0.93 (0.61-1.44) | 0.93 (0.63-1.39) |
| **Incorporating prior GLM classes** | 0.93 (0.60-1.44) | 1.05 (0.73-1.52) |
| **ΔHbA1c>0.5%; ΔBW>2 kg; ΔSBP>2 mm Hg** |  |  |
| **Without prior GLM classes** | 0.86 (0.53-1.41) | 0.85 (0.54-1.34) |
| **Incorporating prior GLM classes** | 0.74 (0.40-1.35) | 0.93 (0.61-1.43) |
| **HbA1c≤7.0%; ΔBW≥3%; SBP <140 mm Hg** |  |  |
| **Without prior GLM classes** | 0.70 (0.41-1.19) | 0.67 (0.37-1.22) |
| **Incorporating prior GLM classes** | 0.56 (0.29-1.07) | 0.75 (0.37-1.55) |

**Table S3.** Adjustment for follow-up duration. The primary MVA and PSM analysis (table 2 of the main manuscript) as well as the sensitivity analysis (^2^) described in Table S2 were further adjusted for follow-up duration in days. The pooled OR (with 95% C.I.) for each composite endpoint was obtained from the 5 imputed datasets and calculated for patients who received dapagliflozin versus those who received GLP-1RA.

| **Outcome** | **MVA** | **PSM** | **IPW^2^** |
| --- | --- | --- | --- |
| **Any reduction in HbA1c, BW, and SBP** | 0.91 (0.64-1.30) | 0.92 (0.59-1.40) | 1.05 (0.47-1.38) |
| **ΔHbA1c>0.5%; ΔBW>2 kg; ΔSBP>2 mm Hg** | 0.83 (0.53-1.29) | 0.73 (0.40-1.30) | 0.93 (0.31-1.75) |
| **HbA1c≤7.0%; ΔBW≥3%; SBP <140 mm Hg** | 0.65 (0.40-1.04) | 0.56 (0.29-1.10) | 0.76 (0.35-1.62) |

**Figure S1**. Concomitant change in medication prescription. The percentage of patients with a change in glucose lowering medications or other medications at the time they initiated dapagliflozin and GLP-1RA. Positive values indicate an increase in prescription, whereas negative values indicate a reduction in prescription. *p<0.05 for chi square test.

**Figure S2. Rebalancing of patient characteristics after propensity score matching**. The graph shows the standardized difference (STD) for each variable calculated in the dataset before (blue) and after (red) propensity score matching (PSM). A STD < 0.10 (dashed line) is indicative of a good match between groups.

BMI, body mass index. SBP, systolic blood pressure. BDP, diastolic blood pressure. FPG, fasting plasma glucose. HDL, high-density cholesterol. LDL, low-density cholesterol. eGFR, estimated glomerular filtration rate. UAER, urinary albumin excretion rate. ACEi, angiotensin converting enzyme inhibitors. ARBs, angiotensin receptor blockers. CCB, calcium channel blockers.

**Figure S3. Common support between the two groups of patients**. Common support refers to the overlap in clinical characteristics between the group of patients who received dapagliflozin and the group of patients who received GLP-1RA. The graph represents the distribution of propensity scores in the two groups of treatment in the first imputed dataset.
